# The predictive value of prognostic immune and nutritional index in esophageal squamous cell carcinoma receiving neoadjuvant immunochemotherapy: a retrospective propensity score matching study

**DOI:** 10.3389/fimmu.2026.1735135

**Published:** 2026-05-21

**Authors:** Xiao Wu, Liang Wang, Xun Yang, Qixun Chen, Jifeng Feng

**Affiliations:** 1 Department of Thoracic Surgery, Zhejiang Cancer Hospital, Hangzhou Institute of Medicine (HIM), Chinese Academy of Sciences, Hangzhou, Zhejiang, China; 2 Key Laboratory Diagnosis and Treatment Technology on Thoracic Oncology, Zhejiang Cancer Hospital, Hangzhou, Zhejiang, China

**Keywords:** disease-free survival, esophageal squamous cell carcinoma, neoadjuvant immunochemotherapy, overall survival, prognosis, prognostic immune and nutritional index

## Abstract

**Background/aim:**

The prognostic immune and nutritional index (PINI) has been increasingly recognized as a significant parameter associated with clinical outcomes in multiple cancers. Nevertheless, its prognostic role in esophageal squamous cell carcinoma (ESCC) undergoing neoadjuvant immunochemotherapy (NICT) remains unexplored.

**Methods:**

This retrospective research enrolled 193 ESCC treated with NICT. The threshold for PINI was determined using the restricted cubic spline (RCS) model. To control for potential confounders, propensity score matching (PSM) was applied. We subsequently assessed the correlations between PINI and both disease-free survival (DFS) and overall survival (OS). Cox proportional hazards regression models were employed to quantify these associations, with results expressed as hazard ratios (HRs) and corresponding 95% confidence intervals (CIs).

**Results:**

The RCS model established an optimal PINI cutoff at 3.320, stratifying all patients into low (n=97) and high (n=96) cohorts. To address potential confounding, PSM generated 64 balanced pairs. Patients in the low cohort demonstrated significantly worse 3-year DFS and OS compared to those with high PINI whether before (DFS: 43.3% vs. 76.0%, P<0.001; OS: 58.8% vs. 84.4%, P<0.001) or after (DFS: 48.4% vs. 68.8%, P = 0.021; OS: 64.1% vs. 81.2%, P = 0.033) PSM. Multivariate analysis confirmed PINI as an independent prognostic index for both DFS (HR = 0.423, 95% CI = 0.257-0.696, P = 0.001) and OS (HR = 0.461, 95% CI = 0.250-0.848, P = 0.013) in the pre-matched cohort. After PSM, PINI remained independently predictive of DFS (HR = 0.543, 95% CI = 0.309-0.955, P = 0.034), though not for OS.

**Conclusion:**

PINI is a significant independent parameter of prognosis, especially in DFS, in patients with ESCC receiving NICT. Incorporating PINI into existing prognostic models may improve long-term risk stratification and assist in personalizing therapeutic strategies.

## Introduction

Esophageal cancer (EC) is a particularly aggressive and anatomically disruptive illness among the various cancers that affect the human gastrointestinal tract (1). In contrast to esophageal squamous cell carcinoma (ESCC), which is dominant in Asian and frequently associated with particular lifestyle exposures, esophageal adenocarcinoma (EAC) has dramatically increased in Western populations, coinciding with the rising prevalence of its known risk factors, such as obesity-driven reflux disease (2, 3). Recently, even with the significant advancements in multidisciplinary managements, such as neoadjuvant chemotherapy (NCT) or neoadjuvant chemoradiotherapy (NCRT), the prognosis for those with disease remains unsatisfactory (4). Even though trimodality therapy—which is exemplified by the CROSS clinical trial—has established itself as the standard treatment, a significant portion of patients still experience disease recurrence, underscoring unresolved issues with overcoming intrinsic resistance and controlling metastasis (5, 6). Due to this clinical conundrum, there has been a paradigm change in favor of using the neoadjuvant phase as a crucial window of opportunity to trigger antitumor immunity. Recently, a growing number of studies suggest that neoadjuvant immunochemotherapy (NICT) is both safe and effective for locally advanced EC (7–9).

Patients are primarily categorized in the current clinical paradigm for EC based on Tumor Node Metastasis (TNM) staging, which serves as a basis for developing later therapy algorithms (10). However, this classification is intrinsically limited because it only takes into account the anatomical extent of the tumor, leaving out important host aspects, including nutritional, inflammatory, and immune status that have a major impact on the prognosis of cancer (11, 12). This means that even among individuals who are diagnosed at the same stage of the disease, significant prognostic heterogeneity typically occurs. Therefore, there is a strong clinical demand for reliable and easily available indicators that can better forecast the behavior of cancer. Neutrophils (NEUs), monocytes (MONs), lymphocytes (LYMs), and platelets (PLTs) are common hematological indices that can be used to easily assess systemic inflammation, a crucial component of carcinogenesis, proliferation, and metastasis (13, 14). In addition to inflammation, nutritional status prior to treatment has been shown to be a significant predictor of cancer prognosis (15). Because of this, composite hematological indices—which combine inflammatory and nutritional data, such as LYM to MON ratio (LMR), PLT to LYM ratio (PLR), NEU to LYM ratio (NLR), systemic inflammation response index (SIRI), and prognostic nutritional index (PNI)—have become popular as useful instruments for improving prognostic classification for a range of cancers (16–18).

The prognostic immune and nutritional index (PINI), a recently developed hematological index integrating systemic inflammatory and nutritional status, has demonstrated superior prognostic predictive ability over several established indicators in colorectal cancer (CRC) (19). This utility has since been validated across several malignancies, including EC (20–23). However, there are still few sensitive hematological indices available to forecast treatment outcomes for EC patients undergoing NICT. Therefore, we hypothesized that in ESCC patients having NICT followed by radical resection, a low pretreatment PINI is linked to decreased disease-free survival (DFS) and overall survival (OS). A propensity score matching (PSM) analysis was also performed to evaluate the usefulness of pretreatment PINI for predicting clinical outcomes in this patient cohort in order to further explore its predictive ability.

## Materials and methods

### Research design and case selection

This retrospective research included patients diagnosed with ESCC who received NICT from 2019 to 2021. According to the Declaration of Helsinki, the study was carried out with ethical approval from Zhejiang Cancer Hospital’s Ethics Committee (IRB2020320). Clinical data and hematological parameters for enrolled cases were obtained from medical records. Exclusion criteria comprised as follow: (1) non-ESCC pathological subtypes; (2) surgery without NICT; (3) failure to achieve radical resection after NICT; (4) incomplete follow-up records or missing essential clinical data; (5) concurrent other additional anticancer therapies; (6) coexisting autoimmune, hematological, or inflammatory disorders; and (7) current or previous diagnosis of other malignant tumors. The patient screening process is illustrated in **Supplementary Figure 1**. The major postoperative complications after surgery following NICT were categorized by the Clavien-Dindo classification (24). Tumor staging was determined based on the 8th edition TNM classification system (10).

### Treatment and follow-up

All patients received two cycles of NICT administered at 21-day intervals prior to surgery. Each treatment cycle consisted of camrelizumab (200 mg, day 1), carboplatin (area under the curve [AUC] = 5 mg/mL/min, day 1), and albumin-bound paclitaxel (120 mg/m², days 1 and 8). Surgical resection via the McKeown or Ivor Lewis approach was scheduled 4–6 weeks after completion of NICT (25). Regarding to adjuvant treatment, at present, no standardized adjuvant therapy was mandated. However, in line with the CheckMate 577 clinical research design, adjuvant immunotherapy was recommended—though not compulsory—particularly for patients with postoperative pathological stages ypT3–T4a and/or ypN1–3 (26). The final follow-up was conducted in December 2024.

### Hematological parameters assessment

Laboratory parameters, such as serum albumin (ALB), MONs, PLTs, LYMs, and NEUs, were extracted from electronic medical records. Peripheral blood samples were obtained from each patient within 1 week before the NICT of their primary tumor. PINI was calculated using the following formula: [ALB (g/dL) × 0.9] − [MONs (/μL) × 0.0007] (19). Other immune- or nutritional-based indices included the PNI, NLR, PLR, SIRI, and LMR. PNI was determined as 10 × ALB (g/dL) + 0.005 × LYMs (/μL) (18). SIRI was calculated as (NEUs × MONs)/LYMs (17); NLR as NEUs/LYMs (16); LMR as LYMs/MONs (16); and PLR as (PLTs/LYMs (16).

### Statistical analysis

Categorical parameters were presented as numbers (percentages). Continuous parameters in non-normally and normally distributed data were presented as median with interquartile range (IQR) and mean with standard deviation (SD), respectively. For group comparisons, the Mann-Whitney U test was used for non-normally distributed continuous data, the Student’s t-test for normally distributed continuous variables, and the Chi-square test for categorical variables. Through an analysis of its non-linear association with survival outcomes, the ideal PINI cutoff was identified using restricted cubic spline (RCS). The RCS model is a powerful tool in the analysis of non-linear dose-effect associations between continuous exposure and outcome (27). Considering the previous literature, using the RCS to determine the cut-off value may have better significance. To reduce the inherent risks of overfitting and optimistic bias in data-driven cut-off selection, additionally, we have also compared the RCS-derived cutoff with other two additional independent methods: receiver operating characteristic (ROC) curve and X-tile. Decision curve analysis (DCA), time-dependent AUC evaluations, and ROC curves were used to examine the clinical value and predictive performance of PINI. In order to equalize baseline characteristics, PSM was then carried out in a 1:1 ratio using nearest-neighbor matching with a caliper width of 0.2. Standardized mean differences (SMD) were calculated before and after PSM, with SMD > 0.1 indicating significant baseline differences between groups. Subgroup analyses were also carried out to explore PINI influence on prognosis (DFS/OS) across different participant groups and comorbid conditions, using a multivariate Cox model. Log-rank tests were used to compare the survival outcomes, which were shown using Kaplan-Meier curves. Independent prognostic parameters for DFS and OS were determined using Cox proportional hazards models; the results were presented as hazard ratios (HRs) and 95% confidence intervals (CIs). In our study, both OS and DFS were calculated from the date of surgery. Specifically, OS was defined as the time from surgery to death from any cause, and DFS was defined as the time from surgery to disease recurrence or death from any cause, whichever occurred first. Statistical significance was defined as P < 0.05. All statistical analyses were conducted using R version 4.1.2.

## Results

### Clinical characteristics of the cohort

The present research included 193 cases diagnosed with ESCC receiving NICT followed by radical resection. There were 23 (11.9%) women and 170 (88.1%) men, and the mean age of all participants was 62.40 ± 7.47 years (range: 45–75 years). Most patients (86.5%, n=167) underwent McKeown procedure, whereas 13.5% (n=26) underwent Ivor Lewis procedure. Concerning the ypT stages, 62 (32.1%) cases had a stage of T0, 60 (31.1%) cases had a stage of T1-2, and 71 (36.8%) cases had a stage of T3-4a. For the ypN stages, 111 (57.5%) patients scored N0, 47 (24.4%) scored N1, 24 (12.4%) scored N2, and 11 (5.7%) patients scored N3. Following surgery, PCR was performed on 60 (31.1%) patients after NICT. With a median of 40 months, the follow-up duration varied from 7 to 54 months. Seventy-eight patients (40.4%) relapsed, and 55 patients (28.5%) died during the follow-up period (**Supplementary Table 1**).

### Prognostic ability of PINI

**Figure 1:**
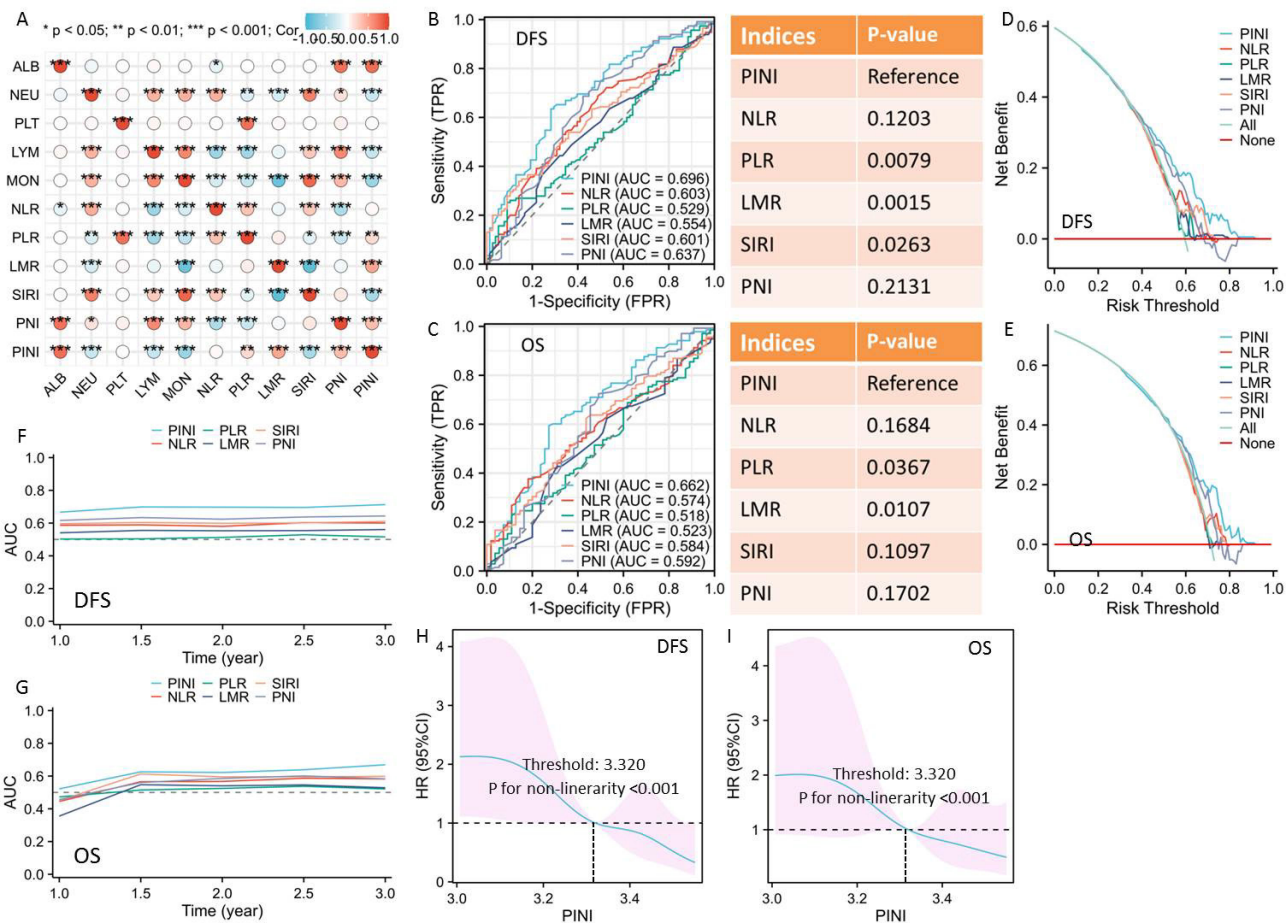
The correlation diagram for each hematological index **(A)**. ROCs for DFS **(B)** and OS **(C)** prediction. DCAs for clinical use of PINI in DFS **(D)** and OS **(E)**. time-dependent AUCs for survival prediction in DFS **(F)** and OS **(G)**. The RCS model for DFS **(H)** and OS **(I)** indicated the proper cut-off value.

The median PINI, as a continuous variable, was 3.32 (Q1-Q3: 3.18-3.52). The correlation diagram for each hematological index is shown in **Figure 1A**. PINI was compared to a number of traditional hematological indicators, such as PLR, LMR, NLR, SIRI, and PNI, in order to assess its predictive ability. When compared to other hematological indices, the ROCs showed that PINI had the greatest AUC (DFS = 0.696 and OS = 0.662), suggesting a stronger predictive power (**Figures 1B, C**). Additionally, the DCAs supported the greater clinical use of PINI in both DFS and OS when compared to other hematological indices (**Figures 1D, E**). In terms of predictive capacity in the time-dependent AUCs, PINI again fared better than the other traditional hematological indices (**Figures 1F, G**). Out of all these hematological indices, therefore, PINI exhibited the best predictive capacity.

### Correlations between PINI and clinical characteristics

There appears to be a non-linear relationship between PINI and DFS/OS, as demonstrated by an RCS analysis. According to **Figures 1H, I**, the RCS model indicated that 3.320 was the proper PINI cut-off value for those with ESCC receiving NICT. Additionally, the X-tile yielded a cutoff of 3.300, and the ROC-derived optimal cutoff (maximizing Youden index) was 3.322 (**Supplementary Figures 2A, B**). The near-identical values across three distinct methods strongly support that the identified threshold is not an artifact of a single analytical approach. All patients were then divided into two groups: low-PINI (n=97) and high-PINI (n=96), with an optimum threshold of 3.320. **Table 1** displays the clinical features categorized by PINI. In comparison to a high PINI, the results showed that a low PINI was substantially linked to a larger tumor length (P = 0.019), a lower PCR rate (P = 0.002), a higher ypT stage (P = 0.013), and a higher ypN stage (P<0.001). In terms of the main postoperative complications, the incidence of respiratory complications was higher in the patients with low-PINI (27.8% vs. 15.6%, P = 0.040; **Supplementary Figure 2C**). However, there was no statistically significant difference with regard to anastomotic fistula (P = 0.379), recurrent laryngeal injury (P = 0.483), or chylous pleural effusion (P = 0.658). Additionally, we matched 64 patient pairings using PSM in order to reduce any potential bias. The clinical characteristics of the low and high groups did not differ significantly (**Table 1**).

**Table 1:** Association between the PINI and clinicopathological features in ESCC receiving NICT before and after PSM.

Parameters	Before PSM				After PSM			
	Low PINI (*n* = 97)	High PINI(*n* = 96)	*P*-value	SMD	Low PINI (*n* = 64)	High PINI (*n* = 64)	*P*-value	SMD
Age, years, Mean ± SD	61.52 ± 6.89	63.29 ± 7.95	0.099	0.240	62.59 ± 6.44	63.45 ± 8.01	0.505	0.119
Sex, *n* (%)			0.845	0.028			0.795	0.046
female	12 (12.37)	11 (11.46)			9 (14.06)	8 (12.50)		
male	85 (87.63)	85 (88.54)			55 (85.94)	56 (87.50)		
BMI, Kg/m^2^, Mean ± SD	21.75 ± 1.81	21.51 ± 1.68	0.333	0.141	21.49 ± 1.74	21.48 ± 1.77	0.985	0.003
Smoking, *n* (%)			0.716	0.052			0.575	0.099
no	27 (27.84)	29 (30.21)			20 (31.25)	23 (35.94)		
yes	70 (72.16)	67 (69.79)			44 (68.75)	41 (64.06)		
Drinking, *n* (%)			0.911	0.016			0.850	0.033
no	29 (29.90)	28 (29.17)			20 (31.25)	21 (32.81)		
yes	68 (70.10)	68 (70.83)			44 (68.75)	43 (67.19)		
Tumor location, *n* (%)			0.122	0.337			0.976	0.068
upper	11 (11.34)	9 (9.38)			7 (10.94)	8 (12.50)		
middle	47 (48.45)	62 (64.58)			39 (60.94)	37 (57.81)		
lower	39 (40.21)	25 (26.04)			18 (28.12)	19 (29.69)		
Surgical method, *n* (%)			0.038	0.303			1.000	0.000
McKeown	79 (81.44)	88 (91.67)			57 (89.06)	57 (89.06)		
Ivor Lewis	18 (18.56)	8 (8.33)			7 (10.94)	7 (10.94)		
Differentiation, *n* (%)			0.287	0.234			0.815	0.043
well	17 (17.53)	26 (27.08)			13 (20.31)	12 (18.75)		
moderate	44 (45.36)	37 (38.54)			27 (42.19)	27 (42.19)		
poor	36 (37.11)	33 (34.38)			24 (37.50)	25 (39.06)		
Vessel invasion, *n* (%)			0.329	0.141			1.000	0.000
negative	80 (82.47)	84 (87.50)			53 (82.81)	53 (82.81)		
positive	17 (17.53)	12 (12.50)			11 (17.19)	11 (17.19)		
Perineural invasion, *n* (%)			0.107	0.234			1.000	0.000
negative	74 (76.29)	82 (85.42)			53 (82.81)	53 (82.81)		
positive	23 (23.71)	14 (14.58)			11 (17.19)	11 (17.19)		
Tumor length, cm, IQR (Q_1_, Q_3_)	2.00 (1.00, 3.10)	1.25 (0.00, 3.00)	0.019	0.294	2.00 (0.00, 3.50)	1.95 (0.00, 3.00)	0.919	0.030
PCR, *n* (%)			0.002	0.467			0.699	0.068
no	77 (79.38)	56 (58.33)			44 (68.75)	46 (71.88)		
yes	20 (20.62)	40 (41.67)			20 (31.25)	18 (28.12)		
Adjuvant therapy, *n* (%)			0.232	0.173			0.828	0.038
no	73 (75.26)	79 (82.29)			51 (79.69)	50 (78.13)		
yes	24 (24.74)	17 (17.71)			13 (20.31)	14 (21.87)		
ypT stage, *n* (%)			0.013	0.416			0.839	0.079
T0	22 (22.68)	40 (41.67)			20 (31.25)	18 (28.12)		
T1-2	34 (35.05)	26 (27.08)			20 (31.25)	22 (34.38)		
T3-4a	41 (42.27)	30 (31.25)			24 (37.50)	24 (37.50)		
ypN stage, *n* (%)			<0.001	0.604			0.857	0.032
N0	42 (43.30)	69 (71.88)			38 (59.38)	39 (60.94)		
N1-3	55 (56.70)	27 (28.12)			26 (40.62)	25 (39.06)		

PINI, prognostic immune and nutritional index; ESCC, esophageal squamous cell carcinoma; NICT, neoadjuvant immunochemotherapy; PSM, propensity score matching; SMD, standardized mean difference; IQR, interquartile range; SD, standard deviation; BMI, body mass index; PCR, pathological complete response; TNM, tumor node metastasis.

### analyse Density curves and subgroup s

The density curves before and after matching were shown in **Supplementary Figure 2D**. The results show that although the sample size is relatively small, the degree of overlap after matching is still quite high. Before matching, the probability density curves of the two groups of propensity scores crossed, indicating that propensity score matching can be performed. The higher the overlap degree of the probability density curves after matching, the better the matching effect. Subgroup analyses were carried out to examine the relationship between PINI and prognosis (DFS/OS) across different categories. The current study indicated that PINI and most subgroup factors did not interact significantly (**Supplementary Figures 3A, B**).

### Comparison DFS and OS grouped by PINI

**Figure 2:**
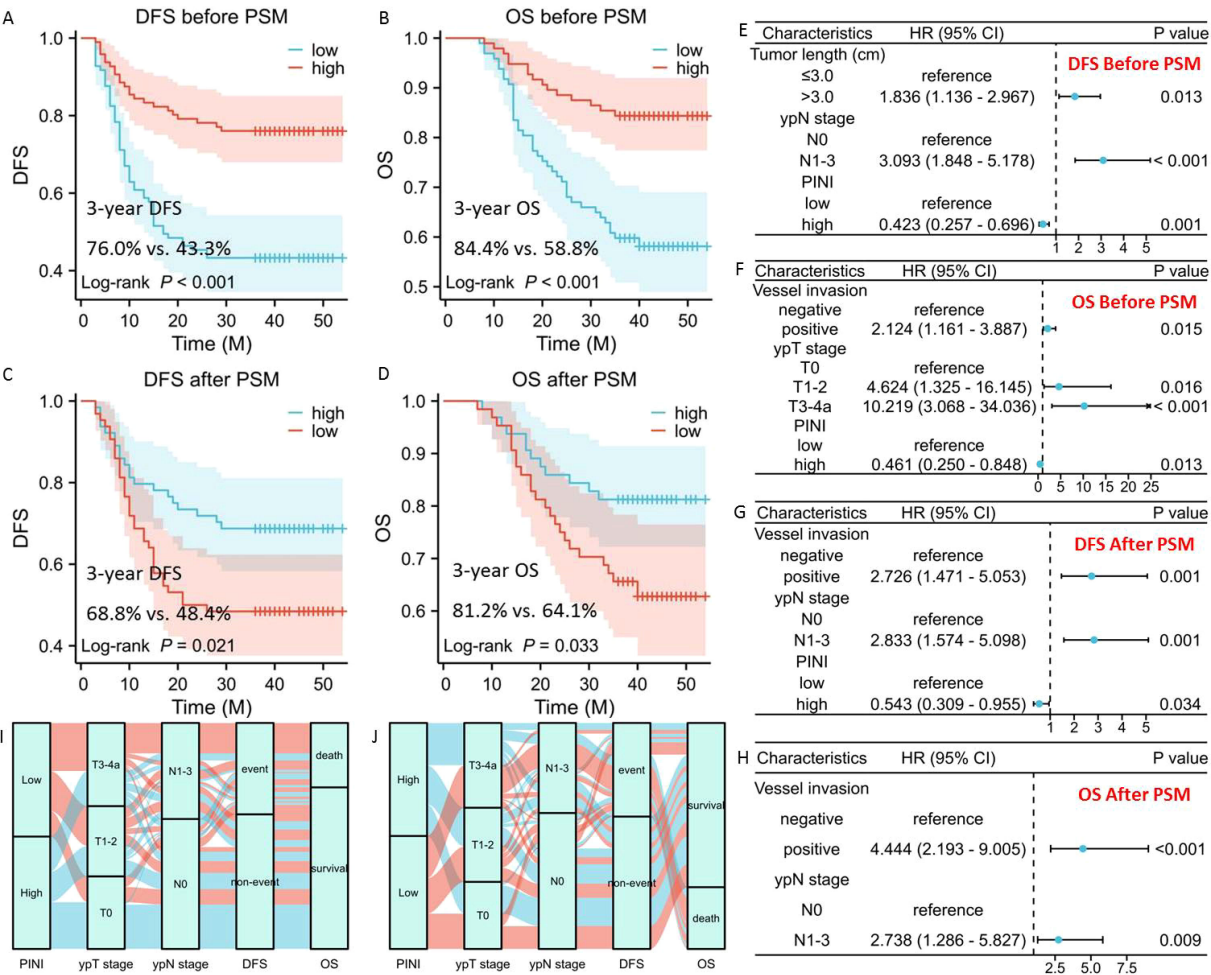
The 3-year DFS **(A)** and OS **(B)** before PSM. The 3-year DFS **(C)** and OS **(D)** after PSM. Multivariate analysis before PSM in DFS **(E)** and OS **(F)**. Multivariate analysis after PSM in DFS **(G)** and OS **(H)**. Sankey diagrams regarding the relationship between PINI and clinical outcomes before **(I)** and after **(J)** PSM.

Prior to PSM, 3-year DFS was considerably worse for patients with low PINI than for those with high PINI (43.3% vs. 76.0%, P<0.001; **Figure 2A**). In a similar vein, patients with high PINI were significantly better off than those with low PINI regarding 3-year OS (P<0.001; **Figure 2B**). The 3-year OS rates for the high PINI group were 84.4%, whereas the low PINI cohort had 58.8%. Additionally, those with low PINI also had a substantially worse 3-year DFS (48.4% vs. 68.8%, P = 0.021; **Figure 2C**) and 3-year OS (64.1% vs. 81.2%, P = 0.033; **Figure 2D**) following PSM than those with high PINI.

### Identification of parameters in DFS and OS

Prior to and following PSM, a number of factors were shown to be substantially correlated with DFS and OS by univariate Cox regression analysis (**Table 2**). PINI was found to be a prognostic parameter associated with DFS and OS in univariate analysis, regardless of whether it occurred before to (DFS: P<0.001 and OS: P<0.001) or following (DFS: P = 0.025 and OS: P = 0.038) PSM. Prior to PSM, multivariate analysis revealed that PINI was an independent predictor of both DFS (HR = 0.423, 95% CI = 0.257-0.696, P = 0.001; **Figure 2E**) and OS (HR = 0.461, 95% CI = 0.250-0.848, P = 0.013; **Figure 2F**). The high PINI cohort decreased the mortality risk by 53.9% and the recurrence risk by 57.7% when compared to the low PINI group. However, following PSM, PINI was demonstrated to be an independent parameter of DFS (HR = 0.543, 95% CI = 0.309-0.955, P = 0.034; **Figure 2G**), but not OS (**Figure 2H**). Sankey diagrams were used to analyze the relationship between PINI and clinical outcomes before to and following PSM. The results indicated that the group with the lower PINI was more likely to die and experience recurrence (**Figures 2I, J**).

**Table 2:** Univariate analyses of prognostic factors associated with DFS and OS in ESCC receiving NICT before and after PSM.

Parameters	Before PSM				After PSM			
	DFS HR (95% CI)	*P*-value	OS HR (95% CI)	*P*-value	DFS HR (95% CI)	*P*-value	OS HR (95% CI)	*P*-value
Age (years, >70 vs. ≤70)	0.848 (0.483-1.489)	0.565	0.502 (0.227-1.110)	0.089	0.902 (0.464-1.752)	0.760	0.413 (0.146-1.171)	0.096
Sex (male vs. female)	0.731 (0.395-1.354)	0.320	0.928 (0.420-2.052)	0.855	0.519 (0.266-1.010)	0.054	0.720 (0.299-1.736)	0.465
BMI (Kg/m^2^, >20 vs. ≤20)	0.752 (0.448-1.261)	0.279	0.972 (0.502-1.881)	0.932	0.790 (0.434-1.436)	0.439	0.899 (0.421-1.919)	0.784
Smoking (yes vs. no)	0.766 (0.480-1.222)	0.264	1.105 (0.611-2.001)	0.741	0.689 (0.399-1.191)	0.182	0.979 (0.487-1.967)	0.952
Drinking (yes vs. no)	1.100 (0.676-1.790)	0.701	1.629 (0.859-3.090)	0.135	1.101 (0.618-1.960)	0.744	1.742 (0.791-3.836)	0.168
Tumor location								
upper	reference		reference		reference		reference	
middle	0.515 (0.262-1.010)	0.053	0.447 (0.209-0.954)	0.037	0.562 (0.255-1.237)	0.152	0.546 (0.217-1.376)	0.199
lower	0.787 (0.394-1.570)	0.496	0.600 (0.273-1.317)	0.203	0.838 (0.364-1.927)	0.677	0.667 (0.246-1.803)	0.424
Surgical method (IL vs. M)	0.896 (0.461-1.740)	0.746	0.890 (0.403-1.968)	0.774	0.995 (0.425-2.327)	0.990	0.963 (0.340-2.729)	0.944
Differentiation								
well	reference		reference		reference		reference	
moderate	1.726 (0.870-3.426)	0.118	2.324 (0.950-5.686)	0.065	1.070 (0.465-2.462)	0.873	1.437 (0.464-4.457)	0.530
poor	2.321 (1.178-4.573)	0.015	2.923 (1.199-7.128)	0.018	2.038 (0.924-4.493)	0.078	2.590 (0.880-7.617)	0.084
Vessel invasion (yes vs. no)	3.196 (1.926-5.303)	<0.001	4.514 (2.956-7.847)	<0.001	4.235 (2.355-7.614)	<0.001	6.269 (3.208-12.249)	<0.001
Perineural invasion (yes vs. no)	2.023 (1.225-3.342)	0.006	3.081 (1.777-5.341)	<0.001	2.834 (1.533-5.239)	0.001	5.160 (2.614-10.184)	<0.001
Tumor length (cm, >3 vs. ≤3)	2.856 (1.809-4.507)	<0.001	3.383 (1.987-5.760)	<0.001	3.087 (1.791-5.320)	<0.001	3.562 (1.832-6.923)	<0.001
PCR (yes vs. no)	0.222 (0.111-0.444)	<0.001	0.105 (0.033-0.336)	<0.001	0.400 (0.195-0.820)	0.012	0.190 (0.058-0.619)	0.006
Adjuvant therapy (yes vs. no)	1.383 (0.839-2.282)	0.776	1.136 (0.609-2.114)	0.690	1.335 (0.725-2.460)	0.354	1.072 (0.487-2.360)	0.863
ypT stage								
T0	reference		reference		reference		reference	
T1-2	2.214 (1.067-4.593)	0.033	5.345 (1.536-18.603)	0.008	1.461 (0.632-3.376)	0.375	2.567 (0.681-9.675)	0.164
T3-4a	5.436 (2.809-10.518)	<0.001	15.016 (4.631-48.693)	<0.001	3.764 (1.784-7.945)	0.001	8.173 (2.458-27.169)	0.001
ypN stage (N1–3 vs. N0)	4.437 (2.737-7.194)	<0.001	5.652 (3.029-10.546)	<0.001	3.393 (1.943-5.926)	<0.001	4.026 (1.969-8.232)	<0.001
PINI (High vs. low)	0.334 (0.205-0.545)	<0.001	0.324 (0.179-0.587)	<0.001	0.529 (0.303-0.923)	0.025	0.477 (0.237-0.959)	0.038

DFS, disease-free survival; OS, overall survival; ESCC, esophageal squamous cell carcinoma; NICT, neoadjuvant immunochemotherapy; PSM, propensity score matching; HR, hazard ratio; CI, confidence interval; BMI, body mass index; IL/M, Ivor Lewis/ McKeown; PCR, pathological complete response; TNM, tumor node metastasis; PINI, prognostic immune and nutritional index.

## Discussion

The utility of hematological indicators lies in their accessibility, cost-effectiveness, reliable accuracy, and high acceptance. The PINI has drawn increasing interest for its involvement in tumorigenesis, metastasis, and antitumor immunity. In this retrospective analysis of ESCC patients treated with NICT, elevated PINI was a significant predictor of improved DFS and OS. In addition to providing new opportunities for individualized therapy, a methodical integration of PINI with accepted clinicopathological characteristics validates its predictive value in this patient group. The pre-matched cohort’s 3-year DFS and 3-year OS outcomes were confirmed to be independently predicted by PINI. Following PSM, its independent predictive value was sustained for 3-year DFS, but not for 3-year OS. All of these results suggested that PINI is a possible parameter to guide nutritional-immune support and improve treatment approaches.

In colorectal cancer (CRC), the PINI first showed better prognostic prediction power than a number of recognized hematological indicators (19). Its predictive usefulness across various stages of CRC has been repeatedly reaffirmed by subsequent investigations (28, 29). Beyond CRC, the prognostic utility of PINI has been validated in other malignancies, including EC (20–23). Although PINI has been identified as a risk factor for EC in previous studies, our current investigation identifies a number of unique features. First, case data from earlier research sometimes covered more than 10 years and included a variety of disease kinds, including ESCC and EAC—factors that potentially affect results. Our analysis, on the other hand, concentrates on a more recent and uniform group of patients, which may provide empirical data that is more in line with current treatment patterns, such as NICT. Second, methodological variances in statistical analysis, underlying tumor heterogeneity, and demographic variety may all contribute to changes in the ideal PINI cut-off values among studies. Our study used the RCS curve to find the ideal threshold in order to improve statistical rigor and provide a more data-driven approach. Third, there is a substantial correlation between PINI and TNM stage, as shown by both our current findings and prior publications. This suggests that disparities in staging could distort survival differences ascribed to PINI. In order to overcome this, we used PSM to reduce confounding factor interference and increase the validity of our findings.

Although numerous inflammatory and nutritional prognostic markers have been reported in various cancers, the PINI defined by ALB and MON offers several distinct advantages that may explain its superior prognostic value. Firstly, the unique mathematical construction of PINI—a linear subtraction formula—warrants attention. Unlike conventional indices that rely on division or multiplication, the PINI formula directly captures the balance between two host factors that exert opposing influences on prognosis. By subtracting the detrimental MON component from the beneficial ALB component, the PINI formula explicitly quantifies this competitive equilibrium, yielding a net score that reflects the overall host anti-tumor capacity. In a large cohort of 4,535 CRC patients, Jung et al. (19) systematically evaluated all possible combinatorial equations among seven hematological parameters and identified the ALB-MON combination as the most powerful prognostic expression, with the PINI achieving the highest concordance indices for prognosis. Secondly, both components of PINI (ALB and MON) possess unique biological relevance to clinical response, rendering them more informative than other hematological indices. The most prevalent plasma protein produced by the liver, ALB, has long been used as a standard indicator of nutritional status. However, its intimate connection to inflammatory processes has come to light more and more in recent years (30). Inflammation is believed to cause hypoalbuminemia through mechanisms such as decreased ALB half-life and increased capillary permeability, which results in ALB leakage (31). Therefore, hypoalbuminemia not only reflects the host’s systemic inflammation but also hinders the body’s ability to respond correctly to stressors such as chemotherapy or surgery, which ultimately reduces longevity and quality of life (32). In a cohort of patients with EC receiving NCT, pretreatment ALB was the only marker that independently predicted pathological response to NCT (33). Furthermore, emerging evidence identifies pretreatment ALB as a relevant prognostic indicator for immune checkpoint inhibitor (ICI) outcomes across multiple solid tumors, with low ALB levels consistently associated with shorter survival in those receiving ICIs (34). MON, a key component of white blood cells, plays a pivotal role in mediating inflammatory responses. Despite their ability to produce tumoricidal factors, MONs frequently experience functional reprogramming in the tumor microenvironment, which results in the loss of their antitumor potential (35). Instead, they differentiate into tumor-associated macrophages, which facilitate cancer progression via immunosuppression, angiogenesis, and enhancement of tumor invasion (36). A recent meta-analysis evaluating 155 eligible clinical studies demonstrated that MON-related markers are robust predictors of ICI efficacy (37). Tumor monocyte content (TMC) obtained from pretreatment transcriptome deconvolution has been identified as an unexpected ICI-specific predictive index of improved survival in EC patients (38). Additionally, recent research has reported that unique immune nutritional scores that incorporate MON-related indicators accurately predict pathological responses to NICT (39). By integrating ALB and MON, therefore, pretreatment PINI captures two orthogonal yet complementary dimensions of host physiology that are mechanistically linked to treatment response.

PINI has the potential to be a useful tool for pretreatment risk assessment in ESCC patients undergoing NICT because of its simplicity and dual reflection of nutritional and inflammatory status. By helping to identify patients who would not otherwise be considered high-risk based on traditional clinical factors, it could support customized perioperative interventions in clinical practice. Firstly, patients with a low pretreatment PINI may benefit from rigorous preoperative nutritional optimization, including enteral or immune-enhancing formulations, because PINI is a composite measure that reflects both nutritional and inflammatory status (40). Moreover, as up to 80% of EC patients suffer from malnutrition, which greatly reduces treatment tolerance and clinical outcomes, implementing focused nutritional treatments during the perioperative phase is now seen as an essential part of multimodal cancer care (41). Secondly, a potential strategy is multimodal prehabilitation, which combines physical activity, nutritional intervention, and psychological support. Multimodal prehabilitation can lower the frequency of postoperative complications in EC (42). Thirdly, for patients exhibiting a low PINI and a suboptimal response to NICT, more aggressive postoperative management strategies may be justified. Since the prognosis of these patients is relatively poor, necessary postoperative therapy may be beneficial to them. However, following PSM, pretreatment PINI was demonstrated to be an independent parameter of DFS, but not OS. On the one hand, the sample size was reduced after PSM, and OS events are typically fewer than DFS events because OS requires death from any cause, whereas DFS includes recurrence, second primary cancers, or death—outcomes that occur earlier and more frequently. In our matched cohort, the number of OS events was notably smaller compared to DFS events, which may have limited the statistical power to detect a modest but clinically relevant effect of PINI on OS. On the other hand, the median follow-up time in our cohort was not long enough, which may be sufficient to capture most recurrences but insufficient to fully capture OS differences. Therefore, longer follow-up duration may be needed to translate recurrence risk into mortality benefit. The persistent association with DFS suggests that PINI may serve as a useful marker for recurrence risk stratification, potentially identifying patients who could benefit from closer surveillance. However, given the lack of association with OS, PINI should not be used to guide decisions about ESCC receiving NICT.

The current study has a number of limitations that should be carefully considered. Firstly, the retrospective design introduces potential selection bias, attributable to its single-center nature, limited cohort size, and non-randomized allocation of subjects. Nevertheless, by balancing baseline characteristics among comparable groups, PSM managed to partially mitigate these biases and improve the scientific validity of our findings. One notable limitation of this study is the substantial reduction in sample size following PSM. Although PSM effectively balanced observed covariates between the two groups based on density curves and subgroup analyses, the matching process resulted in a relatively small matched sample, which may limit the statistical power to detect meaningful treatment effects. Moreover, a reduced sample size may restrict the generalizability of the findings, as the matched sample may no longer be fully representative of the broader target population. Therefore, the results should be interpreted with caution, and future studies with larger sample sizes are warranted to validate these findings. Secondly, the study was carried out in a Chinese population that had a high ESCC incidence. Although this homogeneity promotes data quality and internal consistency, it also limits the results’ applicability to different ethnic or histological situations. Therefore, it is important to exercise caution when extrapolating these findings and to continue validating them via broad, global populations. Thirdly, depending only on one pretreatment PINI assessment is a significant disadvantage. A one-time assessment might not adequately capture patients’ changing clinical risks due to the dynamic nature of nutritional and inflammatory indices during the perioperative and adjuvant therapy phases. Dynamically monitoring these changes to analyze clinical outcomes and prognosis would be of great significance for guiding ESCC therapy. However, due to current practical constraints, we are unable to perform such dynamic monitoring. In particular, the optimal time points for dynamic assessment and how to differentiate whether changes in PINI are attributable to the effects of NICT versus the tumor response remain to be further elucidated. We are planning to conduct relevant research in this direction and look forward to addressing them in future. Fourthly, PINI is derived from ALB and MON, both of which are influenced by systemic inflammation and nutritional status. Although we applied strict inclusion criteria to exclude those with overt inflammatory conditions, residual confounding by unmeasured inflammatory markers or overall disease burden cannot be fully ruled out. Therefore, the independent predictive value of PINI should be interpreted with caution and warrants further validation in cohorts with more comprehensive inflammatory and nutritional profiling. Fifthly, the cut-off value used in our study differs from those reported in previous studies on PINI in other cancer types. To address this issue, we employed a rigorous approach with RCS and further validated by X-tile and ROC curve. These cross-validated methods yielded a consistent cut-off within our cohort. Nevertheless, this cut-off may not be directly generalizable to other cohorts, different treatment regimens, or varying stages of disease. External validation in independent, multicenter, and larger-sample cohorts is therefore essential before this cut-off can be recommended for routine clinical application. Therefore, to confirm and expand on the current results and conclusions, more extensive, prospective, multi-institutional studies are necessary.

## Conclusions

In summary, for ESCC patients receiving NICT, the PINI is a reliable and independent indicator of survival outcomes, especially DFS. Through the integration of both inflammatory and nutritional parameters, PINI provides a therapeutically valuable tool for risk assessment, allowing for more customized patient care. By incorporating it into current prognostic models, it may be easier to identify high-risk individuals early on who could benefit from specialized supportive measures or increased surveillance. However, future studies should concentrate on PINI’s external validation in a variety of clinical and demographic contexts, as well as its potential to guide individualized treatment plans.

## Data Availability

The original contributions presented in the study are included in the article/**Supplementary Material**. Further inquiries can be directed to the corresponding authors.

## Supplementary material

Supplementary Table 1 Clinical characteristics and hematological indices of ESCC receiving NICT

Supplementary Figure 1 The patient screening process. A total of 193 patients were included, with 97 cases in the low group and 96 cases in the high group. PSM generated 64 balanced pairs.

Supplementary Figure 2 X-tile **(A)** and ROC curve **(B)** for cut-off value. Correlations between PINI and postoperative complications **(C)**. The density curves before and after PSM **(D)**.

Supplementary Figure 3 Forest plots of HRs regarding PINI for DFS **(A)** and OS **(B)** in different subgroups.

## References

[1] Bray F Laversanne M Sung H Ferlay J Siegel RL Soerjomataram I . Global cancer statistics 2022: GLOBOCAN estimates of incidence and mortality worldwide for 36 cancers in 185 countries. CA Cancer J Clin. (2024) 74:229–63. doi: 10.3322/caac.21834. PMID: 38572751

[2] Lander S Lander E Gibson MK . Esophageal cancer: Overview, risk factors, and reasons for the rise. Curr Gastroenterol Rep. (2023) 25:275–9. doi: 10.1007/s11894-023-00899-0. PMID: 37812328

[3] Sheikh M Roshandel G McCormack V Malekzadeh R . Current status and future prospects for esophageal cancer. Cancers (Basel). (2023) 15:765. doi: 10.3390/cancers15030765. PMID: 36765722 PMC9913274

[4] Tsuji T Matsuda S Takeuchi M Kawakubo H Kitagawa Y . Updates of perioperative multidisciplinary treatment for surgically resectable esophageal cancer. Jpn J Clin Oncol. (2023) 53:645–52. doi: 10.1093/jjco/hyad051. PMID: 37282626

[5] Malekzada F Vladimiriov M Leitz M Michel J Nimzewski F Hoeppner J . Neoadjuvant treatment of esophageal cancer: Chemotherapy, chemoradiation, immunotherapy, and future trends of therapy. Innov Surg Sci. (2024) 10:3–9. doi: 10.1515/iss-2023-0005. PMID: 40144785 PMC11934940

[6] Eyck BM van Lanschot JJB Hulshof MCCM van der Wilk BJ Shapiro J van Hagen P . Ten-year outcome of neoadjuvant chemoradiotherapy plus surgery for esophageal cancer: The randomized controlled CROSS trial. J Clin Oncol. (2021) 39:1995–2004. doi: 10.1200/jco.20.03614. PMID: 33891478

[7] Williams KM Banks KC Velotta JB . Novel neoadjuvant immunotherapy treatment and surveillance strategies in resectable esophageal cancer: Innovation leads to improved outcomes. J Thorac Dis. (2025) 17:1802–6. doi: 10.21037/jtd-24-1867. PMID: 40400970 PMC12090161

[8] Li CR Lin MZ Li B Li HT Chen YZ Zheng ZZ . Efficacy and safety of neoadjuvant immunotherapy combined with chemotherapy in patients with resectable esophageal squamous cell carcinoma: A systematic review and meta-analysis. Transl Cancer Res. (2025) 14:3373–99. doi: 10.21037/tcr-2024-2631. PMID: 40687234 PMC12268882

[9] Zhang J Zhao P Xu R Han L Chen W Zhang Y . Comparison of the efficacy and safety of perioperative immunochemotherapeutic strategies for locally advanced esophageal cancer: A systematic review and network meta-analysis. Front Immunol. (2024) 15:1478377. doi: 10.3389/fimmu.2024.1478377. PMID: 39712027 PMC11659204

[10] Rice TW Ishwaran H Hofstetter WL Kelsen DP Apperson-Hansen C Blackstone EH . Recommendations for pathologic staging (pTNM) of cancer of the esophagus and esophagogastric junction for the 8th edition AJCC/UICC staging manuals. Dis Esophagus. (2016) 29:897–905. doi: 10.1111/dote.12533. PMID: 27905172 PMC5591444

[11] Xie Y Liu F Wu Y Zhu Y Jiang Y Wu Q . Inflammation in cancer: Therapeutic opportunities from new insights. Mol Cancer. (2025) 24:51. doi: 10.1186/s12943-025-02243-8. PMID: 39994787 PMC11849313

[12] Ahmedah HT Basheer HA Almazari I Amawi KF . Introduction to nutrition and cancer. Cancer Treat Res. (2024) 191:1–32. doi: 10.1007/978-3-031-55622-7_1. PMID: 39133402

[13] Ravindranathan D Master VA Bilen MA . Inflammatory markers in cancer immunotherapy. Biol (Basel). (2021) 10:325. doi: 10.3390/biology10040325. PMID: 33924623 PMC8069970

[14] Zhang YY Liu FH Wang YL Liu JX Wu L Qin Y . Associations between peripheral whole blood cell counts derived indexes and cancer prognosis: An umbrella review of meta-analyses of cohort studies. Crit Rev Oncol Hematol. (2024) 204:104525. doi: 10.1016/j.critrevonc.2024.104525. PMID: 39370059

[15] Hamaker ME Oosterlaan F van Huis LH Thielen N Vondeling A van den Bos F . Nutritional status and interventions for patients with cancer - A systematic review. J Geriatr Oncol. (2021) 12:6–21. doi: 10.1016/j.jgo.2020.06.020. PMID: 32616384

[16] Tan S Zheng Q Zhang W Zhou M Xia C Feng W . Prognostic value of inflammatory markers NLR, PLR, and LMR in gastric cancer patients treated with immune checkpoint inhibitors: A meta-analysis and systematic review. Front Immunol. (2024) 15:1408700. doi: 10.3389/fimmu.2024.1408700. PMID: 39050856 PMC11266030

[17] Wu Z Zhang Z Gu C . Prognostic and clinicopathological impact of systemic inflammation response index (SIRI) on patients with esophageal cancer: A meta-analysis. Syst Rev. (2025) 14:104. doi: 10.1186/s13643-025-02847-7. PMID: 40346701 PMC12063246

[18] Zheng Z Zhu H Cai H . Preoperative prognostic nutritional index predict survival in patients with resectable esophageal squamous cell carcinoma. Front Nutr. (2022) 9:824839. doi: 10.3389/fnut.2022.824839. PMID: 35495910 PMC9043690

[19] Jung SH Hao J Shivakumar M Nam Y Kim J Kim MJ . Development and validation of a novel strong prognostic index for colon cancer through a robust combination of laboratory features for systemic inflammation: A prognostic immune nutritional index. Br J Cancer. (2022) 126:1539–47. doi: 10.1038/s41416-022-01767-w. PMID: 35249104 PMC9130221

[20] Shibutani M Kashiwagi S Fukuoka T Iseki Y Kasashima H Maeda K . Significance of the prognostic immune and nutritional index in patients with stage I-III colorectal cancer. Cancer Diagn Progn. (2023) 3:354–9. doi: 10.21873/cdp.10223. PMID: 37168960 PMC10165384

[21] Aoyama T Hashimoto I Maezawa Y Hara K Kato A Kazama K . The prognostic immune and nutritional indices are independent prognostic factors for esophageal cancer patients who receive curative treatment. Anticancer Res. (2024) 44:2185–92. doi: 10.21873/anticanres.17025. PMID: 38677744

[22] Aoyama T Hashimoto I Maezawa Y Hara K Tamagawa A Cho H . The clinical impact of the prognostic immune and nutritional index in gastric cancer patients who received curative treatment. Anticancer Res. (2024) 44:2231–8. doi: 10.21873/anticanres.17030. PMID: 38677750

[23] Yamashita S Okugawa Y Kitajima T Ieki H Shimamura M Ma R . Association between prognostic immune nutritional index and disease-free survival in adults with esophageal cancer following surgery: A retrospective cohort study. JPEN J Parenter Enteral Nutr. (2025) 49:497–506. doi: 10.1002/jpen.2740. PMID: 40051181 PMC12053140

[24] Dindo D Demartines N Clavien PA . Classification of surgical complications: A new proposal with evaluation in a cohort of 6336 patients and results of a survey. Ann Surg. (2004) 240:205–13. doi: 10.1097/01.sla.0000133083.54934.ae PMC1360123 15273542

[25] Sabra MJ Alwatari YA Wolfe LG Xu A Kaplan BJ Cassano AD . Ivor Lewis vs Mckeown esophagectomy: Analysis of operative outcomes from the ACS NSQIP database. Gen Thorac Cardiovasc Surg. (2020) 68:370–9. doi: 10.1007/s11748-020-01290-w. PMID: 31933140

[26] Kelly RJ Ajani JA Kuzdzal J Zander T Van Cutsem E Piessen G . Adjuvant nivolumab in resected esophageal or gastroesophageal junction cancer. N Engl J Med. (2021) 384:1191–203. doi: 10.1056/nejmoa2032125. PMID: 33789008

[27] Desquilbet L Mariotti F . Dose-response analyses using restricted cubic spline functions in public health research. Stat Med. (2010) 29:1037–57. doi: 10.1002/sim.3841. PMID: 20087875

[28] Xie H Wei L Liu M Liang Y Yuan G Gao S . Prognostic significance of preoperative prognostic immune and nutritional index in patients with stage I-III colorectal cancer. BMC Cancer. (2022) 22:1316. doi: 10.1186/s12885-022-10405-w. PMID: 36522702 PMC9756500

[29] Kayikcioglu E Iscan G . A novel prognostic index for metastatic colon cancer: The prognostic immune nutritional index. Cureus. (2023) 15:e33808. doi: 10.7759/cureus.33808. PMID: 36819360 PMC9931376

[30] Rizo-Téllez SA Sekheri M Filep JG . C-reactive protein: A target for therapy to reduce inflammation. Front Immunol. (2023) 14:1237729. doi: 10.3389/fimmu.2023.1237729. PMID: 37564640 PMC10410079

[31] Soeters PB Wolfe RR Shenkin A . Hypoalbuminemia: Pathogenesis and clinical significance. JPEN J Parenter Enteral Nutr. (2019) 43:181–93. doi: 10.1002/jpen.1451. PMID: 30288759 PMC7379941

[32] Kang B Zhao ZQ Liu XY Cheng YX Tao W Wei ZQ . Effect of hypoalbuminemia on short-term outcomes after colorectal cancer surgery: A propensity score matching analysis. Front Nutr. (2022) 9:925086. doi: 10.3389/fnut.2022.925086. PMID: 36105581 PMC9464913

[33] Noble F Hopkins J Curtis N Kelly JJ Bailey IS Byrne JP . The role of systemic inflammatory and nutritional blood-borne markers in predicting response to neoadjuvant chemotherapy and survival in oesophagogastric cancer. Med Oncol. (2013) 30:596. doi: 10.1007/s12032-013-0596-6. PMID: 23690267

[34] Saal J Ellinger J Ritter M Brossart P Hölzel M Klümper N . Pretreatment albumin is a prognostic and predictive biomarker for response to atezolizumab across solid tumors. Clin Transl Immunol. (2023) 12:e1472. doi: 10.1002/cti2.1472. PMID: 37946873 PMC10632074

[35] Olingy CE Dinh HQ Hedrick CC . Monocyte heterogeneity and functions in cancer. J Leukoc Biol. (2019) 106:309–22. doi: 10.1002/jlb.4ri0818-311r. PMID: 30776148 PMC6658332

[36] Patysheva M Frolova A Larionova I Afanas'ev S Tarasova A Cherdyntseva N . Monocyte programming by cancer therapy. Front Immunol. (2022) 13:994319. doi: 10.3389/fimmu.2022.994319. PMID: 36341366 PMC9631446

[37] Ezdoglian A Tsang-A-Sjoe M Khodadust F Burchell G Jansen G de Gruijl T . Monocyte-related markers as predictors of immune checkpoint inhibitor efficacy and immune-related adverse events: A systematic review and meta-analysis. Cancer Metastasis Rev. (2025) 44:35. doi: 10.1007/s10555-025-10246-6. PMID: 39982537 PMC11845441

[38] Carroll TM Chadwick JA Owen RP White MJ Kaplinsky J Peneva I . Tumor monocyte content predicts immunochemotherapy outcomes in esophageal adenocarcinoma. Cancer Cell. (2023) 41:1222–1241.e7. doi: 10.1016/j.ccell.2023.06.006. PMID: 37433281 PMC11913779

[39] Hamai Y Emi M Ibuki Y Kurokawa T Yoshikawa T Ohsawa M . Ability of blood cell parameters to predict clinical outcomes of nivolumab monotherapy in advanced esophageal squamous cell carcinoma. Onco Targets Ther. (2023) 16:263–73. doi: 10.2147/ott.s404926. PMID: 37065776 PMC10103711

[40] Fujiwara Y Endo S Higashida M Kubota H Yoshimatsu K Ueno T . The prognostic significance of preoperative nutritional/inflammatory markers and clinicopathological features in resectable esophagectomy patients: Possibility of nutritional intervention. Esophagus. (2023) 20:234–45. doi: 10.1007/s10388-022-00961-2. PMID: 36327058

[41] Hussein M Mohamed T Mubarak FA Morad F Naser M Humaira A . Nutritional support and immunonutrition in esophageal cancer - From perioperative care to long-term survivorship: A review. Biomol BioMed. (2025) 26:1251–9. doi: 10.17305/bb.2025.13328. PMID: 41334710 PMC13021243

[42] Shen Y Cong Z Ge Q Huang H Wei W Wang C . Effect of nutrition-based prehabilitation on the postoperative outcomes of patients with esophagogastric cancer undergoing surgery: A systematic review and meta-analysis. Cancer Med. (2024) 13:e70023. doi: 10.1002/cam4.70023. PMID: 39001679 PMC11245637

