# Supplementary material for: The predictive value of prognostic immune and nutritional index in esophageal squamous cell carcinoma receiving neoadjuvant immunochemotherapy: a retrospective propensity score matching study
Source: Front Immunol. 2026 May 21;17:1735135. doi: 10.3389/fimmu.2026.1735135 (PMC13233711; doi:10.3389/fimmu.2026.1735135)
Supplement: Supplementary Table 1 — Clinical characteristics and hematological indices of ESCC receiving NICT [file Table1.docx]

**Table S1** **Clinical characteristics and hematological indices of ESCC receiving NICT**

| Clinical characteristics | Total (n=193) | Hematological indices | Total (n=193) |
| --- | --- | --- | --- |
| Age (mean ± SD, range, years)  Sex (female/male, n, %)  BMI (mean ± SD, range, Kg/m^2^)  Smoking history (yes/no, n, %)  Drinking history (yes/no, n, %)  Tumor location (upper/middle/lower, n, %)  Surgical method (McKeown/Ivor Lewis, n, %)  Differentiation (well/moderate/poor, n, %)  Vessel invasion (negative/positive, n, %)  Perineural invasion (negative/positive, n, %)  Tumor length (IQR, Q1-Q3, cm)  Adjuvant therapy (yes/no, n, %)  PCR (yes/no, n, %)  ypT stage (T0/T1-T2/T3-T4a, n, %) | 62.40 ± 7.47 (45-75)  23 (11.9)/170 (88.1)  21.63 ± 1.75 (17.96-26.22)  137 (71.0)/56 (29.0)  136 (70.5)/57 (29.5)  20 (10.4)/109 (56.4)/64 (33.2)  167 (86.5)/26 (13.5)  43 (22.3)/81 (42.0)/69 (35.7)  29 (15.0)/164 (85.0)  37 (19.2)/156 (80.8)  1.90 (0, 3.0)  41 (21.2)/152 (78.8)  60 (31.1)/133 (68.9)  62 (32.1)/60 (31.1)/71 (36.8) | Albumin (IQR, Q1-Q3, g/dL)  Neutrophil (IQR, Q1-Q3, 10^9^/L)  Platelet (IQR, Q1-Q3, 10^9^/L)  Monocyte (IQR, Q1-Q3, 10^9^/L)  Lymphocyte (IQR, Q1-Q3, 10^9^/L)  NLR (IQR, Q1-Q3)  PLR (IQR, Q1-Q3)  LMR (IQR, Q1-Q3)  SIRI (IQR, Q1-Q3)  PNI (IQR, Q1-Q3)  PINI (IQR, Q1-Q3) | 4.12 (3.98-4.20)  3.90 (3.50-4.20)  195.0 (166.0-224.5)  0.50 (0.40-0.60)  1.40 (1.30-1.60)  2.75 (2.50-3.07)  136.9 (115.3-166.5)  2.80 (2.43-3.20)  1.43 (1.14-1.69)  48.2 (46.9-49.5)  3.32 (3.18-3.52) |
| ypN stage (N0/N1/N2/N3, n, %) | 111 (57.5)/47 (24.4)/ 24(12.4)/11 (5.7) |  |  |

**Abbreviation:** ESCC: esophageal squamous cell carcinoma; NICT: neoadjuvant immunochemotherapy; SD: standard deviation; BMI: body mass index; IQR: interquartile range; PCR: pathological complete response; TNM: tumor node metastasis; NLR: neutrophil to lymphocyte ratio; PLR: platelet to lymphocyte ratio; LMR: lymphocyte to monocyte ratio; SIRI: systemic inflammation response index; PNI: prognostic nutritional index; PINI: prognostic immune and nutritional index.
