# Supplementary figures and images for: The predictive value of prognostic immune and nutritional index in esophageal squamous cell carcinoma receiving neoadjuvant immunochemotherapy: a retrospective propensity score matching study
Source: Front Immunol. 2026 May 21;17:1735135. doi: 10.3389/fimmu.2026.1735135 (PMC13233711; doi:10.3389/fimmu.2026.1735135)

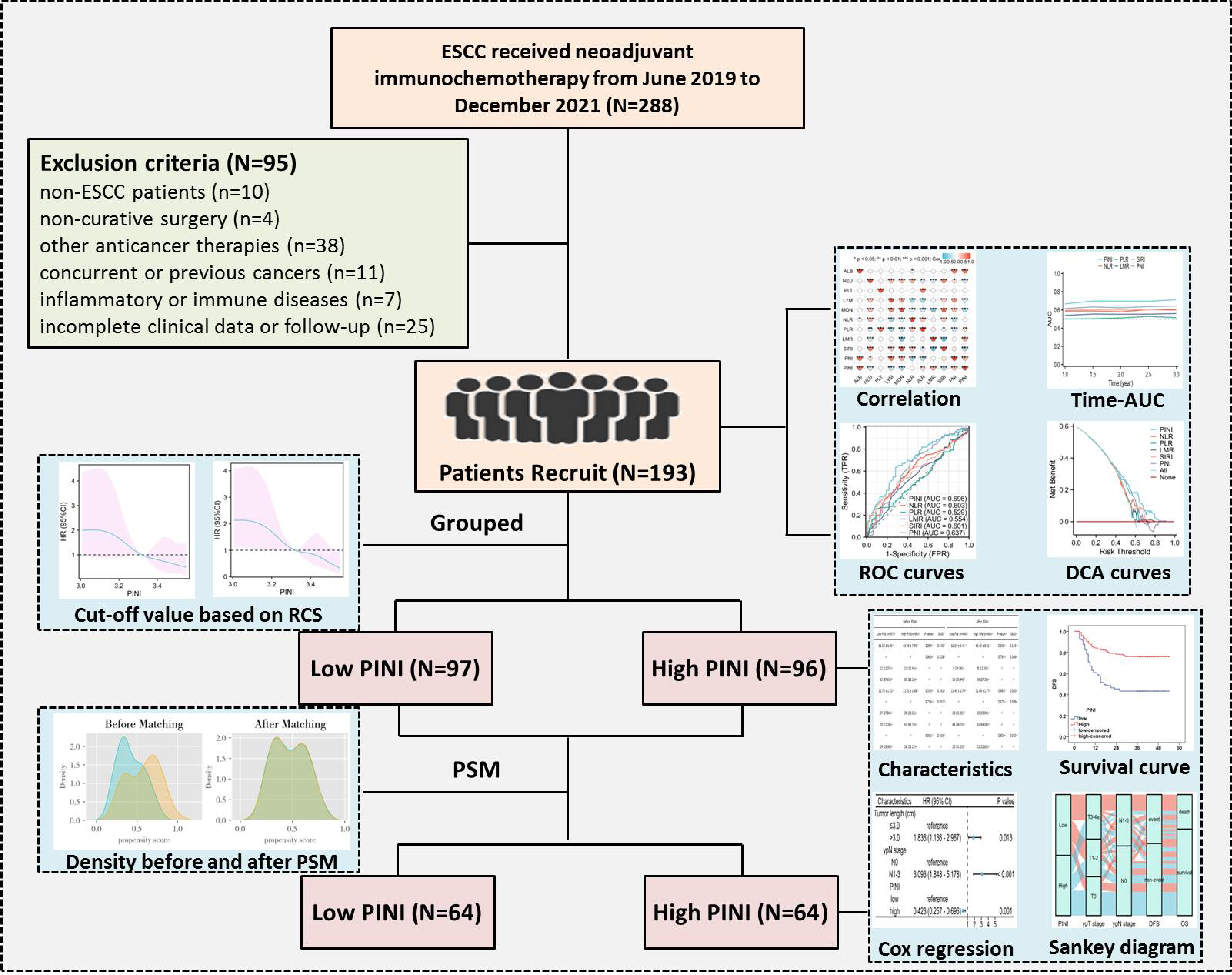

Supplement: Supplementary Figure 1 — The patient screening process. A total of 193 patients were included, with 97 cases in the low group and 96 cases in the high group. PSM generated 64 balanced pairs. [file Image1.jpeg]

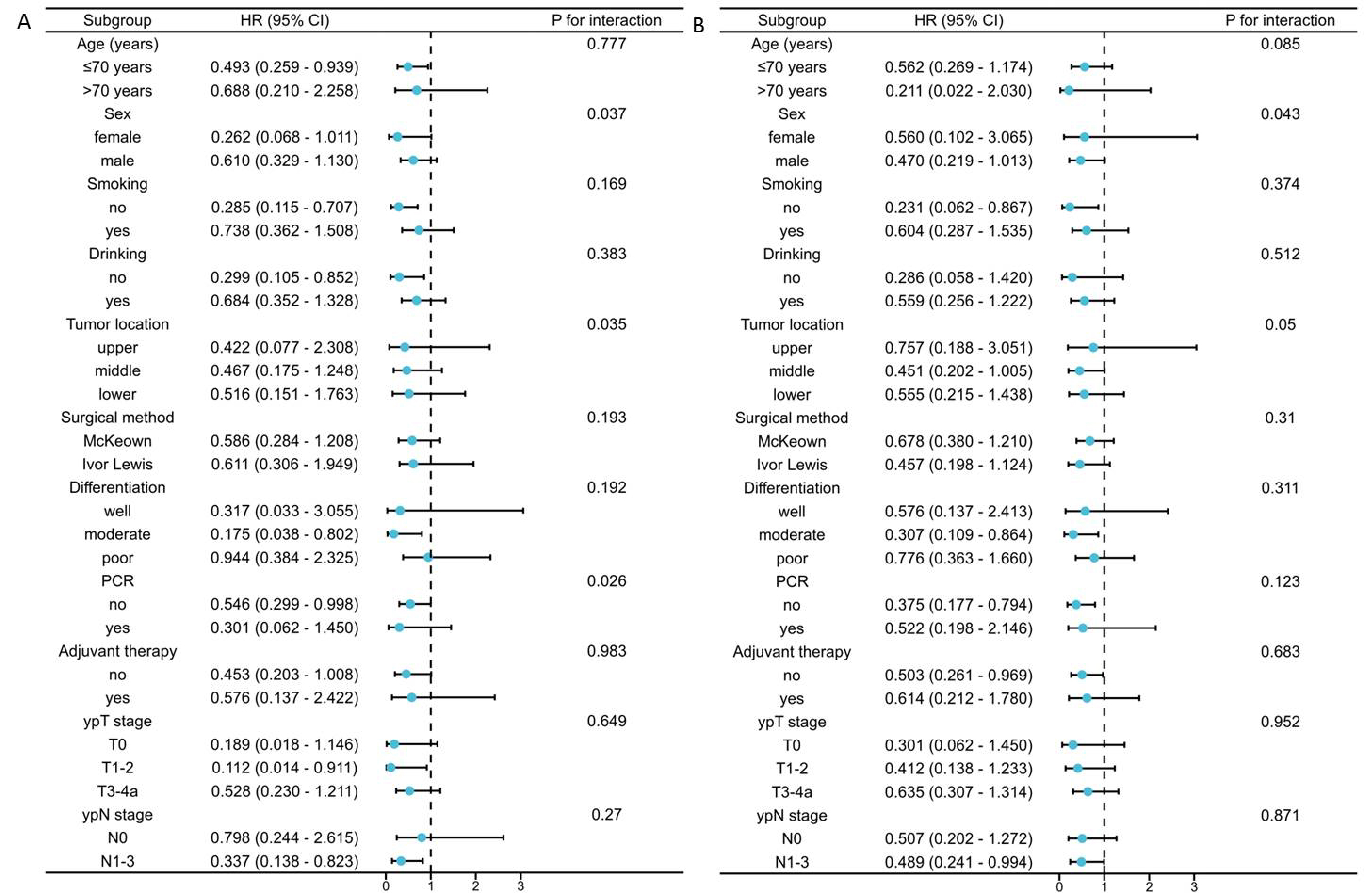

Supplement: Supplementary Figure 2 — X-tile (A) and ROC curve (B) for cut-off value. Correlations between PINI and postoperative complications (C). The density curves before and after PSM (D). [file Image2.jpeg]

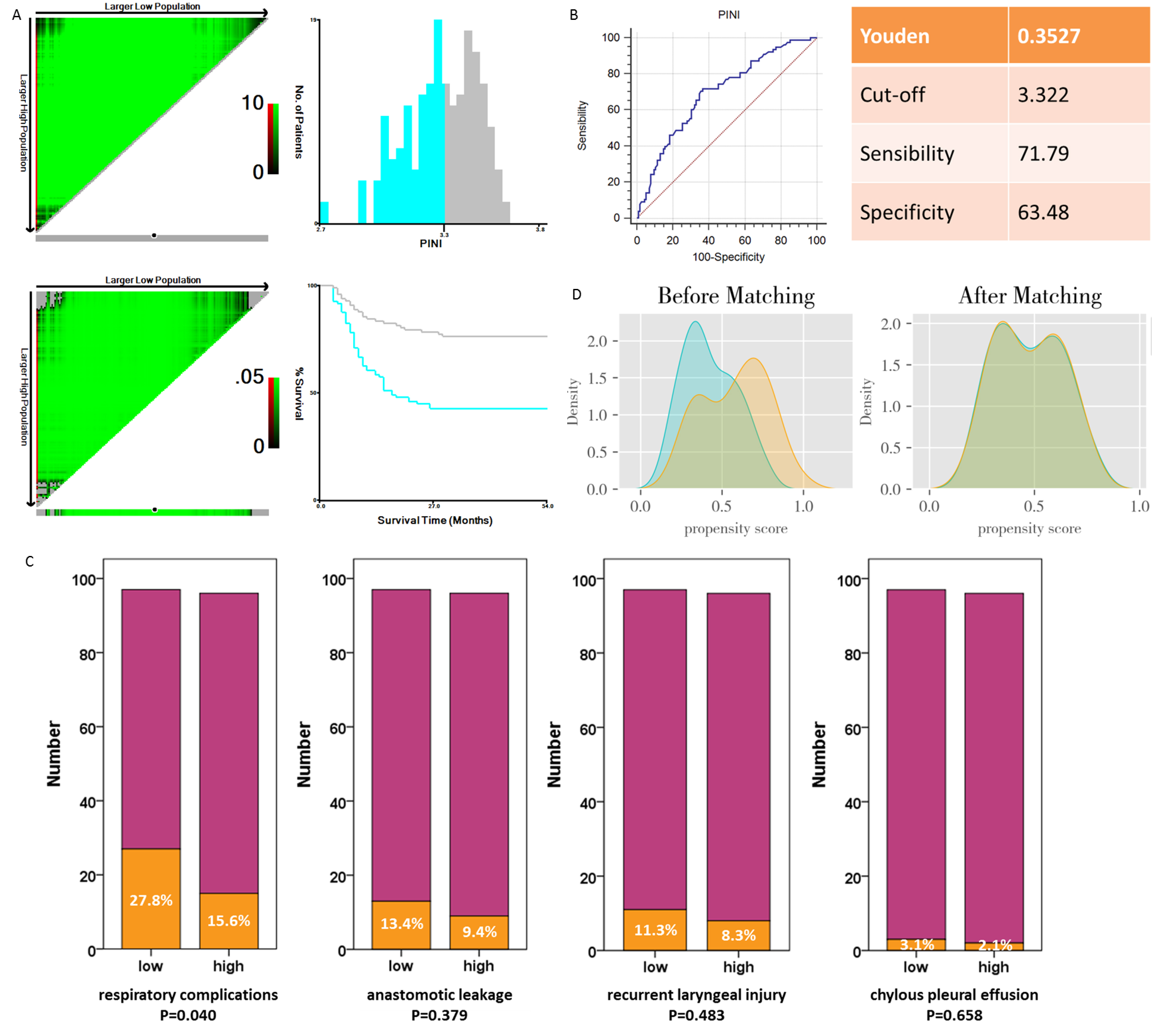

Supplement: Supplementary Figure 3 — Forest plots of HRs regarding PINI for DFS (A) and OS (B) in different subgroups. [file Image3.jpeg]
